# Gestational arsenic exposure induces site-specific DNA hypomethylation in active retrotransposon subfamilies in offspring sperm in mice

**DOI:** 10.1186/s13072-020-00375-3

**Published:** 2020-12-02

**Authors:** Keiko Nohara, Kazuhiko Nakabayashi, Kazuyuki Okamura, Takehiro Suzuki, Shigekatsu Suzuki, Kenichiro Hata

**Affiliations:** 1 Center for Health and Environmental Risk Research, National Institute for Environmental Studies, Tsukuba, 305-8506 Japan; 2 Department of Maternal-Fetal Biology, National Center for Child Health and Development, Tokyo, 157-8535 Japan; 3 Center for Environmental Biology and Ecosystem Studies, National Institute for Environmental Studies, Tsukuba, 305-8506 Japan

**Keywords:** Sperm, DNA methylation, Retrotransposon, LINE, LTR, Gestational exposure, Arsenic

## Abstract

**Background:**

Environmental impacts on a fetus can disrupt germ cell development leading to epimutations in mature germ cells. Paternal inheritance of adverse health effects through sperm epigenomes, including DNA methylomes, has been recognized in human and animal studies. However, the impacts of gestational exposure to a variety of environmental factors on the germ cell epigenomes are not fully investigated. Arsenic, a naturally occurring contaminant, is one of the most concerning environmental chemicals, that is causing serious health problems, including an increase in cancer, in highly contaminated areas worldwide. We previously showed that gestational arsenic exposure of pregnant C3H mice paternally induces hepatic tumor increase in the second generation (F2). In the present study, we have investigated the F1 sperm DNA methylomes genome-widely by one-base resolution analysis using a reduced representation bisulfite sequencing (RRBS) method.

**Results:**

We have clarified that gestational arsenic exposure increases hypomethylated cytosines in all the chromosomes and they are significantly overrepresented in the retrotransposon LINEs and LTRs, predominantly in the intergenic regions. Closer analyses of detailed annotated DNA sequences showed that hypomethylated cytosines are especially accumulated in the promoter regions of the active full-length L1MdA subfamily in LINEs, and 5′LTRs of the active IAPE subfamily in LTRs. This is the first report that has identified the specific positions of methylomes altered in the retrotransposon elements by environmental exposure, by genome-wide methylome analysis.

**Conclusion:**

Lowered DNA methylation potentially enhances L1MdA retrotransposition and cryptic promoter activity of 5′LTR for coding genes and non-coding RNAs. The present study has illuminated the environmental impacts on sperm DNA methylome establishment that can lead to augmented retrotransposon activities in germ cells and can cause harmful effects in the following generation.

## Background

Environment affects human health. Particularly, a fetus is vulnerable to such environmental impacts [1, 2]. The impacts on the fetus (F1) can induce long-term adverse effects after birth and are paternally and/or maternally inherited in their offspring (F2) and occasionally also in the subsequent generations in humans and model animals. Epigenomes, such as DNA methylomes, histone modifications and small RNAs of germ cells take pivotal roles in the inheritance, and paternal inheritance is exclusively attributed to the epigenomes of sperm [2, 3].

The gestational period is critical for the development of germ cells in fetuses, as they need to undergo epigenetic reprogramming to continue development [4–7]. In mice, primordial germ cells (PGCs) that can differentiate into sperm and ova are generated from epiblast and reach fetal gonads at around gestational day (GD) 7.5. The DNA methylomes that were acquired from epiblast were once largely eliminated by GD13.5, which enables the cells to develop into germ cells. In males, the germ cells undergo epigenetic reprogramming and reestablish DNA methylomes by GD17.5. After birth, the germ cells differentiate from spermatogonia to mature sperm and methylome patterns are duplicated [8]. The methylomes of mature sperm need to be accurately reestablished to organize differentiation-specific gene expressions in cooperation with other epigenomes. On the other hand, gestational environmental exposure has been reported to affect epigenetic reprogramming leading to epimutations in mature germ cells and intergenerational and transgenerational outcomes [1, 2, 9]. However, the impacts of gestational exposure to a variety of environmental factors on the germ cell epigenomes are not fully clarified on molecular bases.

Arsenic is one of the most concerning environmental chemicals causing serious health problems. Inorganic arsenic is a component of earth’s crust and millions of people in the areas where arsenic levels are high, such as certain areas in China, Bangladesh, India, Chile, Mexico and the United States, are suffering from chronic exposure to inorganic arsenic mainly through drinking water and food [10, 11]. In addition to chronic exposure, gestational and early age exposure to arsenic is growing concerns [12]. Health problems reported to be caused by such exposures include cancers and cardiovascular, respiratory, metabolic, reproductive and neurological disorders [10–14]. As a characteristic feature, arsenic has been known for more than two decades to induce DNA methylation changes [10, 15, 16]. In our previous study, we found that arsenic exposure from GD 8 to 18 of pregnant C3H mice, whose males are prone to stochastically develop spontaneous hepatic tumors, increased hepatic tumor incidence in F2 males later in life [17]. We also found that the tumor increase is paternally inherited to the F2 [17]. DNA methylome analyses of F2 hepatic tumors identified genes whose expressions are closely associated with methylation changes and are involved in tumor development in the arsenic group [18]. These results prompted us to investigate whether gestational arsenic exposure induces any alteration in DNA methylation in the F1 sperm. In order to understand paternal inheritance of environmental impacts, clarification of DNA methylome changes as well as other epigenetic alterations in male germ cells is crucial.

DNA methylome analyses generally give priority to specific genomic regions, mainly the transcription start sites (TSSs) of protein-coding genes, as they are functionally important. Those analyses have so far obtained novel findings in a few studies, such as methylation changes of metabolism-related genes in F1 sperm by gestational undernutrition of F0 mothers [19] and imprinting gene hypomethylation in F1 sperm by gestational exposure to a pesticide metabolite [20]. On the other hand, mammalian genomes have acquired huge amounts of retrotransposons by autonomous amplification of these elements in the long history of evolution. The mouse genome contains close to 40% of transposon-derived sequences, including about 20% of the long interspersed nuclear elements (LINEs) and 10% of the long-terminal repeats (LTRs)-derived elements [21–23]. Although a large extent of those retrotransposons is inactivated by extensive rearrangements and truncations, some subfamilies hold transposition activities by escaping truncation and some hold cryptic promoter activities even in the truncated forms [22, 23]. Mice genome contains 2000–3000 full-length LINE elements, per individual, that are still transcriptionally active and potentially affect host genomes [24, 25]. Among LTRs, IAPE is one of the most transcriptionally active LTR subfamilies and is well studied [23, 26–28]. Retrotransposition of transposable elements in germ cells are potentially harmful for the germ cells themselves or the subsequent generations [24]. Retrotransposon-dependent promoter activities are implicated in gene functions by affecting the expression of coding genes and non-coding RNAs. DNA hypermethylation of the regulatory regions of these elements are essentially involved in the suppression of retrotransposition activities and promoter activities [4–7]. However, closer analysis has not been done on the impacts of environment on region-specific DNA methylomes of retrotransposon subfamilies in the sperm.

In the present study, in order to seek whether and how gestational arsenic exposure affects methylomes of F1 sperm, we investigated the DNA methylation changes including repeat sequences with particular attention to the position of each differentially methylated cytosine by one-base resolution analyses using a reduced representation bisulfite sequencing (RRBS) measurement. As a result, we have found novel significant effects of gestational arsenic exposure on F1 sperm methylomes.

## Results

### F1 sperm DNA methylomes are less divergent among individuals but are increased in hypomethylated cytosines by gestational arsenic exposure

**Fig. 1:**
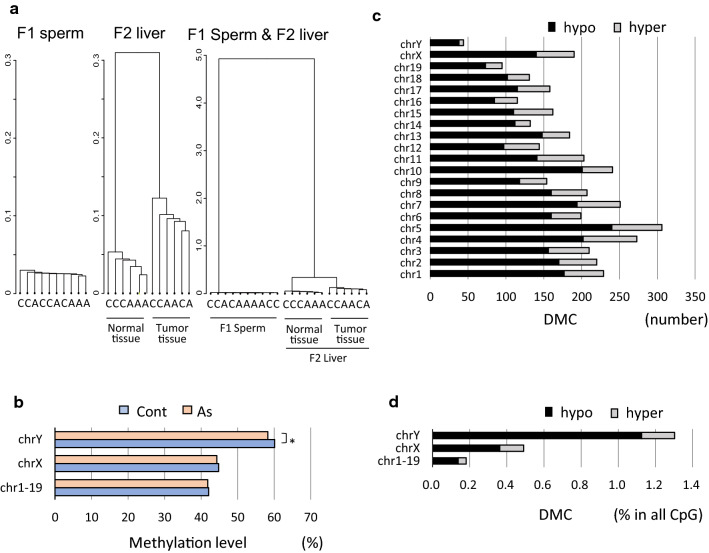
Profiles of sperm DNA methylomes from the control and arsenic F1 mice. **a** Cluster analysis of methylomes of F1 sperm and normal and tumor tissues from F2 livers. **b** Average methylation levels of all CpGs in each chromosome in the F1 sperm. The differences in the methylation levels between the control and arsenic groups were tested by Student *t* test (*p < 0.05). The number (**c**) and % of hypo/hyperDMCs in all CpGs (**d**) in each chromosome in the F1 sperm

DNA samples were prepared from purified sperm of individual F1 mice born to five control dams and five gestationally arsenite exposed dams, respectively, and subjected to RRBS analysis. The number of all CpGs identified was 2,039,237 as described in the “Methods” section. The methylomes of F1 sperm were found to be less divergent with minor individual differences compared to those of normal livers and hepatic tumor tissues that were analyzed in our previous studies [18, 29] (Fig. 1a). The average methylation levels of all CpG sites were 42.13 ± 0.04% in the control group and 41.90 ± 0.06% in the arsenic group. They were slightly lowered in autosomes and chromosome X (chrX) and statistically significantly lowered in chrY of the arsenic group compared with the control group (Fig. 1b).

On the other hand, we found a remarkable feature of the effects by gestational arsenite exposure on F1 sperm by detecting hypodifferentially methylated cytosines (hypoDMCs) and hyperdifferentially methylated cytosines (hyperDMCs) that are hypo- or hypermethylated with not lower than 10% methylation differences in the arsenic group compared to the control group. We identified 2,958 hypoDMCs and 905 hyperDMCs. The predominance of hypoDMCs was observed over all the chromosomes (Fig. 1c). Particularly, chrY contained a higher percentage of hypoDMCs and was followed by chrX in abundant hypoDMCs (Fig. 1d). These features were confirmed in another independently conducted experiment (Additional file 1: Figure S1). These results showed that gestational arsenic exposure has consistent effects on offspring sperm.

### HypoDMCs are enriched in LINEs and LTRs in arsenic F1 sperm

**Fig. 2:**
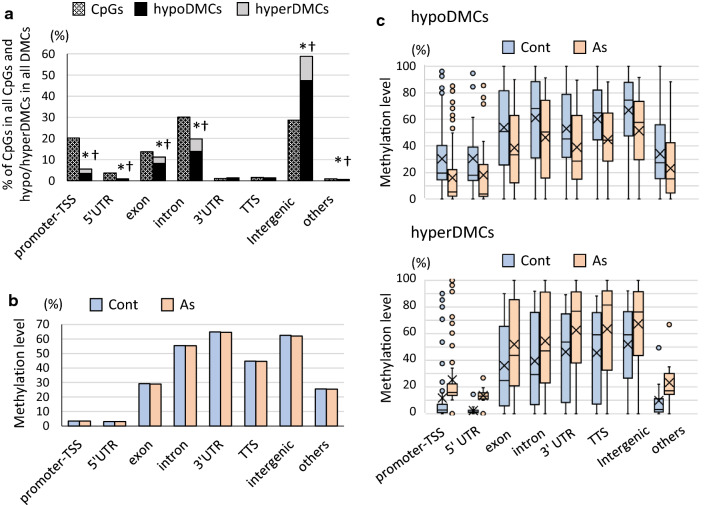
Annotation of all CpGs and DMCs in all the genomic regions and their DNA methylation levels. **a** Distribution (%) of annotated genomic regions among all CpGs (hatched bars) and among DMCs including hypoDMCs (black bars) and hyperDMCs (gray bars). Occurrence of DMCs (hypo and hyperDMCs) and hypoDMCs in each region was assessed using Fisher’s exact test and *p* < 0.001 was marked with * and †, respectively. **b** Average methylation levels of all CpGs in the individual genomic regions. **c** Box-and-whisker plots of methylation levels of hypo/hyperDMCs in the individual genomic regions. Crosses and bars in the box represent average and median values, respectively, and dots indicate outliers

To determine the distribution of DMCs in genomic regions, we annotated all CpGs using the HOMER (Hypergeometric Optimization of Motif EnRichment) software. The major parts of CpGs were annotated with intron, intergenic, promoter-TSS, exon and 5′-UTR in descending order of percentage according to the ‘annotation’ of the software (Fig. 2a, Additional file 1: Table S1A, B). On the other hand, DMCs and particularly hypoDMCs, were overrepresented in intergenic and underrepresented in genic regions including promoter-TSS, 5′UTR, intron and exon regions, indicating methylomes of genic region are refractory to arsenic (Fig. 2a). The average methylation levels of CpGs were lower in promoter-TSS and 5′UTR (Fig. 2b) consistent with our previous analyses on liver tissues [18] and with general aspects of genomic DNA methylomes. In a comparison between the control and arsenic groups, the average methylation levels of CpGs were slightly lower in the arsenic group in all the regions (Fig. 2b). Although the average methylation levels of promoter-TSS and 5′UTR are less than 5%, these regions still contain CpGs that have higher methylation levels in the control and that are hypoDMCs in the arsenic group (Fig. 2c). The CpG sites having relatively higher methylation levels in the control tended to become hypoDMCs in general (Fig. 2c).

**Fig. 3:**
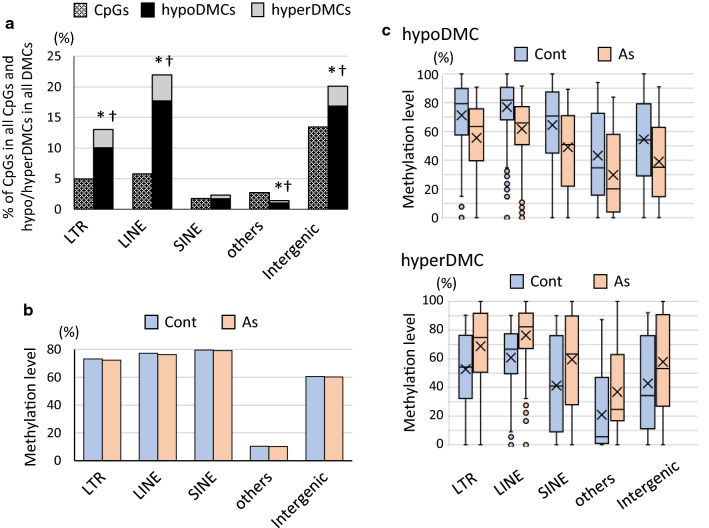
Detailed annotation of all CpGs and DMCs in the intergenic region and their methylation levels. **a** All CpGs (hatched bars) and DMCs including hypoDMCs (black bars) and hyperDMCs (gray bars) in the intergenic assignment shown in Fig. 2a were further annotated according to the detailed annotation of HOMER. Occurrence of DMCs (hypo and hyperDMCs) and hypoDMCs in each assignment was assessed using Fisher’s exact test and *p* < 0.001 was marked with * and †, respectively. **b** Average methylation levels of CpGs in each detailed annotation shown in **a**. **c** Box-and-whisker plots of methylation levels of hypo/hyperDMCs in each detailed annotation shown in **a**. Crosses and bars in the box represent average and median values, respectively, and dots indicate outliers

As predominant DMCs were annotated as intergenic, we further annotated intergenic CpGs and DMCs according to the ‘detailed annotation’ of HOMER. The intergenic includes the detailed annotations: LTR, LINE, SINE, others such as simple repeats DNA transposons, and intergenic, an assignment that is not annotated any further (Additional file 1: Table S1B). DMCs with predominantly hypoDMCs in intergenic were highly overrepresented in LINE and LTR, the retrotransposon elements (Fig. 3a). When the CpGs in all the genomic regions were annotated according to the detailed annotation, the same feature, that is, remarkable overrepresentation of DMCs and hypoDMCs in LINE and LTR, were observed (Additional file 1: Figure S2). The methylation levels of CpGs in LINE and LTR were generally high (Fig. 3b) and relatively higher methylated CpGs in these regions turned out to be hypoDMCs (Fig. 3c).

### HypoDMC enrichment in the promoters of an active LINE subfamily L1MdA in arsenic F1 sperm

**Fig. 4:**
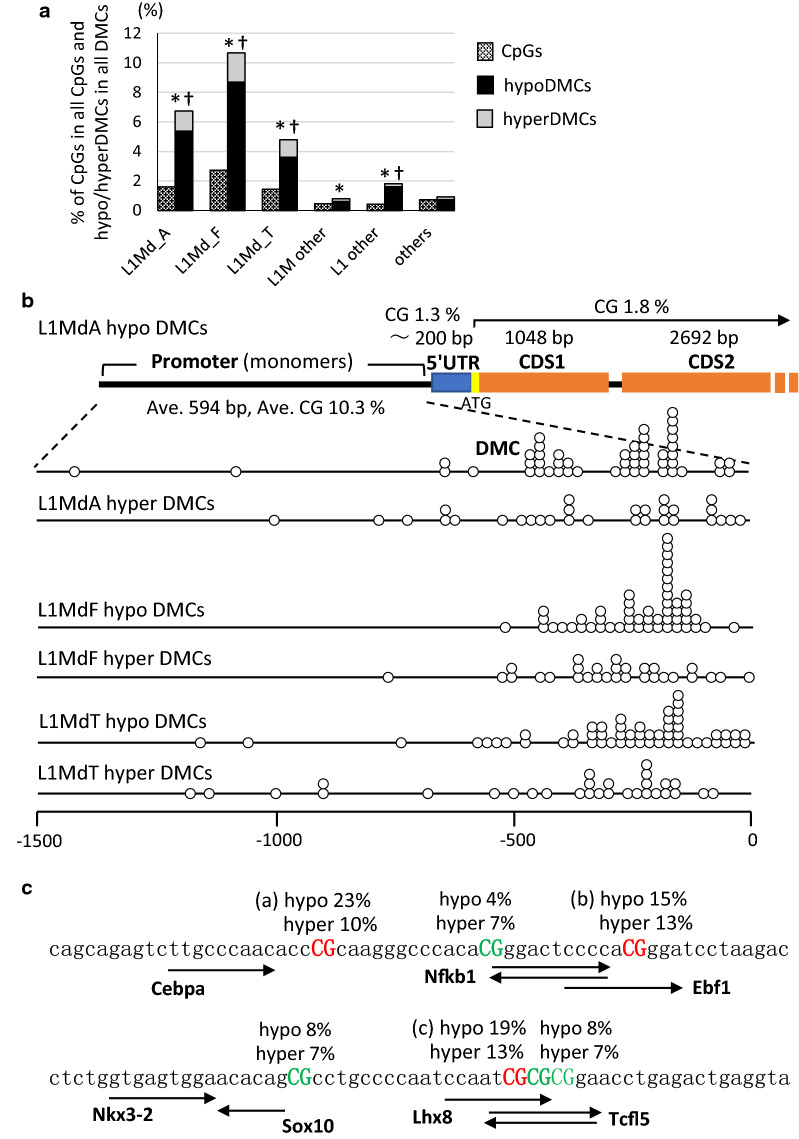
HypoDMCs were overrepresented in an active L1MdA subfamily in LINE. **a** All CpGs (hatched bars) and DMCs including hypoDMCs (black bars) and hyperDMCs (gray bars) in LINE in all the genomic region shown in Additional file 1: Fig. S2 were further classified according to the detailed annotation given by HOMER. Occurrence of DMCs (hypo and hyperDMCs) and hypoDMCs in each region was assessed using Fisher’s exact test and *p* < 0.001 was marked with * and †, respectively. **b** A schematic figure of L1MdA containing hypoDMCs and the positions of hypo/hyperDMCs (dots on the lines) in the promoter regions of full-length L1Md subfamilies. Fifty and 30 sequences were randomly selected from full-length L1MdA, L1MdF and L1MdT containing hypoDMCs and hyperDMCs, respectively, using SeqKit and the positions of DMCs were analyzed. The scheme at the top shows the average lengths of individual domains and % CpGs in the domains in L1MdAs containing hypoDMCs. **c** Transcription factor binding sites in a consensus sequence of L1MdA including hypoDMCs. Highly consistent sequences around the DMC hot spots (**a**–**c** in the figure) were extracted among 50 L1MdAs containing hypoDMCs. Percentages in 50 hypoDMCs and 30 hyperDMCs appearing in the individual CpGs were indicated in the figure

While the majority of retrotransposons are inactive at present, L1MdA, as well as T_F_ and G_F_ subfamilies are transcriptionally active in LINE elements [25, 30, 31]. According to these previous reports, we classified all CpGs and hypo/hyperDMCs in LINE in all the genomic regions shown in Additional file 1: Fig. S2 according to the detailed annotation of HOMER (Additional file 1: Table S1B). L1MdA, L1MdF and L1MdT were the major subfamilies, to which abundant CpGs belong and in which hypoDMCs were statistically significantly overrepresented (Fig. 4a).

Transcriptionally active L1MdA retrotransposons contain full-length elements consisting of a promoter region including a tandem repeat of about 200 bp monomers, typically 203 bp 5′UTR, two open reading frames (ORF1 and ORF2) and 3′UTR. Hypermethylation of the CpG sites in the promoters are essential in suppression of L1 retrotransposition [32–34]. We identified 208 and 52 L1MdA hypoDMCs and hyperDMCs, respectively. Among L1MdA hypoDMCs, 143 (69%) were identified to be full-length elements and considered potentially active. We also identified 336 and 76 L1MdF hypo and hyperDMCs and 139 and 46 L1MdT hypo and hyperDMCs, respectively. The analysis of the position of a randomly selected 50 hypoDMCs and 30 hyperDMCs in full-length elements found that DMCs were exclusively at the promoter regions of L1MdA, L1MdT and L1MdF subfamilies (Fig. 4b).

Seeking causative alterations leading to hypomethylation of specific positions, we searched binding sites of transcription factors in a consensus sequence of L1MdA containing enriched hypo and hyperDMCs (Fig. 4c). There were three CpG sites which frequently become DMCs in the L1MdA sequence (Fig. 4c). They are overlapped by the transcription factor (TF) binding sites of Ebf1, Lhx8 or Tcfl5 and in proximity to the binding sites of Cebpa and Nfkb1 (Fig. 4c). Binding of such transcription factors may suppress DNA methylation or methylome changes affect promoter activity by altering transcription factor binding. While we found 5′-gagactcgagc-3′ in the L1MdA consensus sequence and that is more than 90% identical to AER-like element reported by Lu and Ramos [35], the CpG site in the sequence did not become DMC.

We also assessed whether ORF1 or ORF2 sequences are related to the occurrence of DMCs, however, no phylogenetic relationships were detected between them (Additional file 2: Figure S3 and Additional file 3: Figure S4).

These results suggest that active L1 promoters are sensitive to arsenic exposure during germ cell development and the resulting hypomethylation may lead to an augmentation of the retrotransposition activity.

### HypoDMC enrichment in an active LTR subfamily IAPE in arsenic F1 sperm

**Fig. 5:**
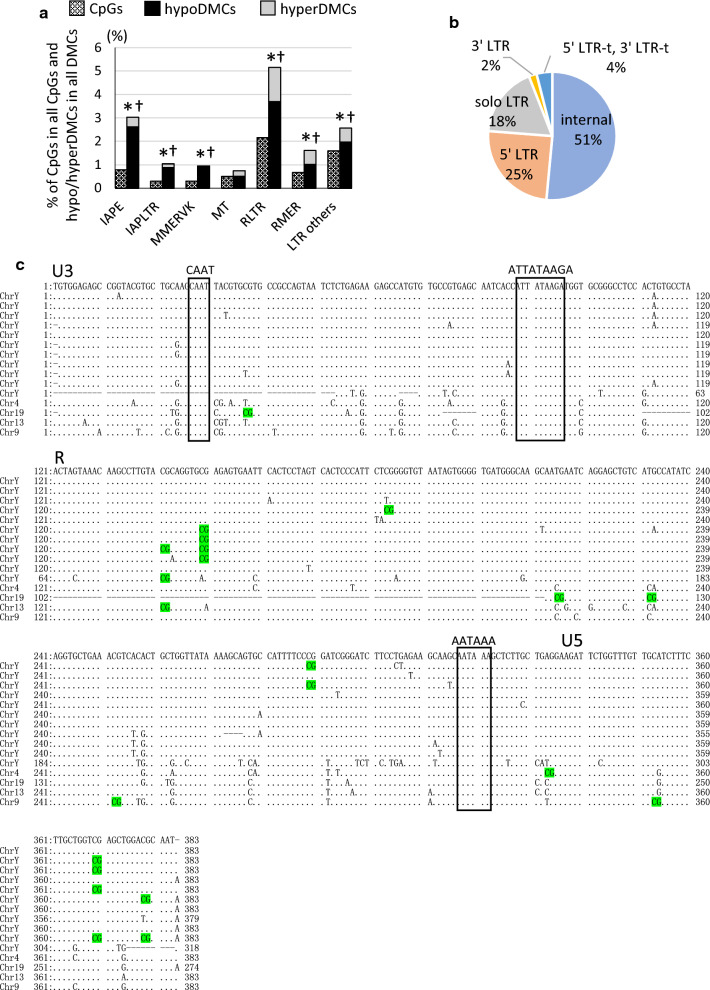
HypoDMCs were overrepresented in an active IAPE subfamily in LTR. **a** All CpGs (hatched bars) and DMCs including hypoDMCs (black bars) and hyperDMCs (gray bars) in LTR in all the genomic regions shown in Additional file 1: Fig. S2 were further classified according to the detailed annotation given by HOMER. Occurrence of DMCs (hypo and hyperDMCs) and hypoDMCs in each region was assessed using Fisher’s exact test and *p* < 0.001 was marked with * and †, respectively. **b** The proportion of IAPE hypoDMC fragments in all IAPE hypoDMCs. **c** The positions of hypoDMCs in the IAPE 5′LTRs. The position of hypoDMCs (colored in green) are shown in 5′LTR sequences containing transcription regulation sequences CAAT and TATA box and polyadenylation site (AATAAA). Dots (.) represents identical base and dashes (–) represents deletion

Detailed annotation of LTRs in all the genomic regions by HOMER showed that RLTR and IAPE subfamilies include higher percentages of hypoDMCs (Fig. 5a). IAPE is among the most transcriptionally active LTR subfamilies [23, 26–28]. IAPs include 5′LTR and 3′LTR elements that identically consist of U3, R and U5 domains flanking internal regions and these LTR fragments contain transcription regulation sequences CAAT and TATA box within the U3 domain and polyadenylation site in the R domain [36]. These LTR fragments possess promoter activity [37–39] which is regulated by DNA methylomes [26]. Methylation within U3 and R domains were reported to be involved in the suppression, while the role of U5 methylomes was not examined [40].

In the present study, we identified 101 hypoDMCs in IAPE and 89 elements were intergenic and the other 12 were intron. We classified those hypoDMCs into 5′LTR, 3′LTR, solo LTR that was generated by rearrangement of 5′LTR and 3′LTR, and internal regions according to Shimosuga et al. [28]. More than half of the IAPE hypoDMCs (50 elements) were classified in the internal region and a quarter (25 elements) were in 5′LTR (Fig. 5b). We assessed the position of hypoDMCs in a consensus sequence of 5′LTR containing CAAT, TATA sequence (5′-ATTATAAGA-3′), and polyadenylation site (AATAAA) and found about half of the hypoDMCs were within U3 and R domains (Fig. 5c). While there were two regions where hypoDMCs were accumulated (5′-CGCAGGTGCG-3′ and 5′-CGAGCTGGACG-3′), no significant overlapping TF binding sites were detected by the JASPER database. These results indicate that gestational arsenic exposure lowers the methylation of IAPE promoters in the sperm which may lead to activation of cryptic promoter activity.

### Overrepresentation of IAPEY in chrY and L1MdA in chrX

**Fig. 6:**
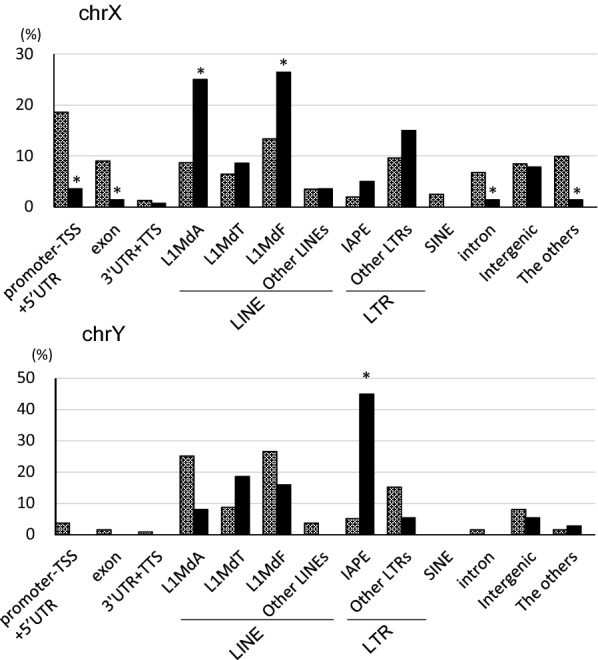
ChrX and ChrY were enriched with IAPE hypoDMCs and with L1MdA and L1MdF hypoDMCs, respectively. All CpGs and hypoDMCs in chrY and chrX in all the genic region were annotated according to the detailed annotation of HOMER. The detailed annotations were grouped as shown in Additional file 1: Table S1B. Percentage among CpGs (hatched bars) and hypoDMCs (black bars) were shown for chrX and chrY, respectively. # promoter indicates promoter-TSS and 5′UTR. Occurrence of hypoDMCs in each region was assessed using Fisher’s exact test and *p* < 0.01 was marked with *

Since chrY in particular, and chrX contained a higher percentage of hypoDMCs (Fig. 1d), we analyzed these hypoDMCs according to the detailed annotation of HOMER. The detailed annotations are grouped as shown in Additional file 1: Table S1B. ChrY contained the smallest number of CpGs which are mostly intergenic (Additional file 1: Figure S5A, B). ChrX was the second smallest in number of CpGs and also contained abundant intergenic assignment (Additional file 1: Figure S5A, B). We identified 38 hypoDMCs in chrY and 140 hypoDMCs in chrX. As shown in Fig. 6, the active retrotransposons IAPE and L1MdA in all the genomic regions were overrepresented in chrY hypoDMCs and chrX hypoDMCs, respectively.

### Methylation levels of paternal imprinting genes in the F1 sperm

While heritable DNA methylation sites are maintained in imprinting gene regions which are evolutionary stable and conserved [41, 42], previous reports indicate that these marks are modestly susceptible to environmental impacts [3, 20]. Thus, we assessed whether hypomethylation of sperm by gestational arsenic exposure affects paternal imprinting regions in the sperm. We calculated the DNA methylation levels of CpG sites in *Gpr1/Zdbf2*, *H19* ICR, *Rasgrf1* and *DLK-Gtl2* IG whose methylation was identified to be maintained at a high level in the sperm [43]. The results showed that the methylation levels of paternally methylated regions were not changed and offered protection from the danger of arsenic exposure (Additional file 1: Figure S6).

### Identification of differentially methylated regions (DMRs) around TSSs

We also analyzed differentially methylated regions that include three or more CpG sites and at least one DMC with ≥ 10% methylation differences in the F1 sperm of the arsenite group, since they are implicated in transcription regulation especially around TSSs. We identified 494 hypoDMRs and 83 hyperDMRs and categorized them according to the detailed annotation as described in the “Methods”. The list of DMRs is available at GSE150650. The most abundant hypoDMRs were detected in the intergenic regions and hyperDMRs were in the LTR regions (Additional file 1: Figure S7). DMRs within TSS ± 2000 bp were selected as shown in Additional file 1: Table S2. The contributions of these DMRs to gene expression await further studies.

## Discussion

It is a relatively recently established notion that DNA methylome changes caused by environmental exposure during germ cell development remain in the sperm after reprogramming and take a role in paternal transmission of the impact of environments [4, 6, 9, 44]. The idea was largely based on accumulating reports on the findings of paternal intergenerational inheritance of environmental impacts and the analyses of sperm methylomes. The present study has added new data of environmental impacts on sperm methylomes by showing intensive accumulation of hypoDMCs in the promoter regions of active full-length L1MdA in LINE and in 5′LTRs of active IAPE in LTR by gestational arsenic exposure. On the other hand, a growing number of studies have reported that impacts of gestational exposure to environmental factors on F1 can be transgenerationally transmitted through the sperm of F2 to subsequent generations even though they are not directly exposed [9, 44, 45]. While the present study showed intergenerational effects of arsenic exposure on methylomes of F1 sperm, further study is needed to clarify whether they are transgenerationally inherited to later generations.

Hypomethylation of promoters of active LINE elements leads to retrotransposition activation. Retrotransposition of transposable elements in germ cells are potentially harmful for the germ cells themselves and the subsequent generations since they disturb critical gene expressions involved in development of germ cells and gametes after fertilization [24]. In the experimental system used in the present study, gestational arsenic exposure led to an increase in hepatic tumor incidence in F2 born to F1 males [17]. L1 mobilization is found to be a common feature of virus-induced and alcoholism-dependent HCC in humans and in a mouse model of human HCC [46, 47]. The consequences of increased hypoDMCs in LINE elements might be clues to elucidate the molecular pathway of paternal transmission of a tumor increase by gestational arsenic exposure.

We have also found that the hypoDMCs accumulate in U3 and R regions of 5′LTRs in the IAPE subfamily. Provided those IAPE fragments have cryptic promoter activity and methylomes of U3 and R regions suppress the activity, IAPE 5′LTR hypoDMCs in the F1 sperm of arsenic group potentially increase the promoter activity. As these DMCs are mainly detected in the intergenic regions, they may be involved in the expression of sperm-specific non-coding RNAs and miRNAs.

The results that gestational arsenic exposure affects DNA methylomes of active LINEs and IAPEs is reminiscent of the piRNA-dependent DNA methylation pathway in sperm, as piRNA-directed de novo methylation during reprogramming is biased toward recently expanding retrotransposon subfamilies, including the L1MdT, L1MdA and IAP subfamilies [48, 49]. Thus, the molecules involved in piRNA-dependent de novo methylation pathway, such as Piwi family proteins, Mili and Miwi2 [50, 51], are considered as potential targets of gestational arsenic exposure.

Although arsenic is a carcinogen, it is generally known to be not a mutagen or a weak mutagen [10]. Our previous study using *gpt* delta transgenic mice which was developed for detection of in vivo mutation showed that exposure to 85 ppm arsenite in drinking water for 3 weeks significantly increased mutation frequency in the livers to 1.10 ± 0.23 × 10^–5^ compared to 0.71 ± 0.06 × 10^–5^ in the control [52]. However, the frequency is much lower than the frequency of DNA methylation changes. Furthermore, germ line is refractory to mutation and mutation frequency in sperm is about two orders lower than somatic cells [53]. Thus, analysis of DNA methylation changes by arsenic would be hardly affected by mutation.

Arsenic compounds interact with biological components such as sulfur compounds, phosphorus and zinc-finger proteins and are known to interact with biological pathways including signal transduction, genotoxicity and epigenetics [10]. However, responsible molecules in a variety of physiological reactions are largely yet to be clarified. Gestationally administered arsenic was detected in the fetus organs such as liver and brain in the same experimental regimen as we used in the present study [54]. Arsenic compounds reaching the fetus gonad may directly or indirectly interfere with factors involved in DNA methylome establishment, such as piRNA pathways as described above, transcription factors such as those shown in Fig. 5c, and histone modifications that direct DNA methylation at reprogramming [7]. Among the transcription factors, Tcfl5 is specifically detected in mouse testis and essential for spermatogenesis [55, 56]. *Ebf1* expression is reported to be affected by a metabolite of inorganic arsenic compound [57]. Studies of these interferences are pivotal to elucidate the mechanism of arsenic-inducing health effects.

In the present study, the percentage of hypoDMCs in chrY was especially higher than those in other chromosomes (Fig. 1d). The number of CpGs identified in the present study was remarkably small (Additional file 1: Fig. S3A) and the most part of CpGs were intergenic (Additional file 1: Fig. S3B). Consistent with such notion, the intergenic region contained higher percentage of IAPEY elements and those elements were prone to be hypoDMCs (Fig. 6), which resulted in the distortion of a higher percentage of hypoDMCs. ChrX is second to chrY in the percentage of hypoDMCs (Fig. 1d). ChrX abounds with more L1 elements than autosomes [21] and L1Md hypoDMCs contribute to the predominance of hypoDMCs in this chromosome.

The present study has illuminated retrotransposon methylomes in the sperm as one of the targets of gestational chemical exposure. The well-known mutant mice which harbor a cryptic IAP promoter upstream of the *Agouti variable yellow* (*A*^*vy*^) locus show variable coat colors depending on the methylation levels of IAP promoter and the phenotype is maternally inherited to the F1 offspring [58]. Gestational exposure of this mice model to an endocrine disruptor bisphenol-A decreases the methylation levels of IAP and accompanying coat color changes in the F1 offspring of exposed dams, while the methylomes of F1 germ cells were not investigated [59]. Direct exposure of humans, model animals and cell lines to a variety of environmental factors reported L1 hypomethylation as the most common observations among the effects on retrotransposons in somatic cells [60]. Although the mechanism of hypomethylation induction in somatic cells by direct exposure and in germ cells during development should be fundamentally different, it is interesting that environment tends to lower the methylation of retrotransposon elements. Environmental impacts may be transgenerationally involved in sperm evolution as DNA methylomes of mature sperm have been reported to have evolutionally expanded hypomethylated regions [61]. As the previous studies reviewed by Miousse et al. [62] were carried out by methods that cannot read precise sequences genome-widely, such as combined bisulfite restriction analysis (COBRA), methylation-sensitive quantitative PCR (MS qPCR) and pyrosequencing, further information is expected by genome-wide sequencing analyses. Regarding the effects of gestational arsenic exposure, it is yet to be clarified whether the hypomethylation of retrotransposon sequences in sperm are inherited and the changes are accumulated when the exposure continues for generations.

## Conclusion

The results of the present study clarified the pressure of gestational environmental exposure on retrotransposon DNA methylomes in the offspring sperm, especially increasing hypomethylation of active LINE and LTR subfamilies, which may lead to intergenerational and transgenerational effects. DNA methylomes of retrotransposons are related to many types of cancers and other diseases [62, 63]. The results of the present study indicate that the contribution of retrotransposon DNA methylome disruption by gestational exposure to a variety of environmental factors remains to be clarified.

## Methods

### Animals and experimental design

Animal treatment was carried out as described previously [17]. Briefly, pregnant F0 C3H/HeN mice were purchased from CLEA Japan (Tokyo, Japan) and given free access to a standard diet (CA-1; CLEA Japan) and tap water (the control group). The arsenite group was given tap water containing 85 ppm sodium arsenite, an inorganic arsenic compound, instead of tap water from gestational day (GD) 8 to 18. F1 males from individual groups were used for isolation of sperm at around 17–19 weeks of age. The mice were handled in a humane manner in accordance with the National Institute for Environmental Studies (NIES) guidelines for animal experiments.

### Isolation of sperm and genomic DNA extraction

A drop of HTF medium (human tubal fluid medium (Irvine Scientific, CA) containing 12.5 mM Hepes and 0.4% BSA) was made in a dish, covered with mineral oil and maintained at 37 °C. Cauda epididymis was put in the oil and a sperm clump was gently taken out from incisions made with scissors and moved to the buffer with a needle. After incubating for 60 min, sperm that swam up around the buffer wall was transferred to ice-cold PBS-0.5% BSA and collected by centrifugation for 2 min at 2000*g* at 4 °C. The pellet was gently broken, treated with lysis buffer (0.1% SDS, 0.5% TritonX100, [64]) for 10 min on ice to lyse somatic cells, and washed with PBS-0.5% BSA.

Five sperm samples (1 × 10^6^ sperm/sample) were obtained from five F1 males of different litters for the control and arsenic groups, respectively. Sperm was lysed in lysis buffer (7.3 mM Tris–HCl (pH 7.5), 7.3 mM NaCl, 18.2 mM EDTA, 1.8% SDS, 1.8% 2-ME, 1.4 mg/ml proteinase K) and DNA was extracted with a phenol–chloroform mixture, precipitated with ethanol, dried and dissolved in 10 mM Tris (pH 7.4).

### DNA methylation analysis by RRBS and determination of differentially methylated cytosines (DMCs) and differentially methylated regions (DMRs)

RRBS libraries were prepared according to Boyle et al. [65] with some modifications. Briefly, 100 ng of genomic DNA was sonicated for one second by a Focused-ultrasonicator (Covaris) before restriction enzyme reaction and processed as described previously [18, 29]. The libraries were sequenced on an Illumina HiSeq X. The total reads obtained were 46.4–63.4 million and total read bases were 6.9–9.5 Gbp. The sequence data are publicly available at Gene Expression Omnibus (GEO) with accession number GSE150650. The sequencing reads were mapped on the mouse reference genome (mm10) using the Bismark program [66] and adapter trimming and quality control were performed using Trim Galore (http://www.bioinformatics.babraham.ac.uk/projects/trim_galore/).

The methylation status of each CpG was calculated using the read.bismark function of a MethylKit package (ver. 0.9.4) [67] on R as described by Okamura et al. [18]. Cluster analyses was performed on the sperm RRBS data obtained in the present study and the liver RRBS data obtained by Matsushita et al. (GEO accession number GSE111420) [29] and Okamura et al. (GEO accession number GSE111420) [18] using MethylKit. The CpG sites that were commonly detected in all ten samples with 10 or more coverage were identified as CpGs. The methylation level of a given CpG in each sample was calculated as (coverage of C/(coverage of C + coverage of T)). Average methylation level was calculated as the mean value of methylation levels of all CpGs concerned. Among CpGs, DMCs and DMRs were selected using a MethylKit package and eDMR package [68] on R, respectively. DMCs were defined as CpGs with q-value ≤ 0.01 by the logistic regression method and ≥ 10% methylation differences between two groups. DMRs were defined as regions which contain ≥ 3 CpGs and at least one DMC and whose methylation levels were different more than 10% with statistically significant differences between the two groups.

### Annotation by HOMER and analysis of retrotransposon subfamilies

The CpGs were annotated using HOMER (http://homer.ucsd.edu/homer/, v4.10) and categorized according to those annotation and detailed annotation assignments of the software. Homer uses UCSC refGene annotation (http://homer.ucsd.edu/homer/ngs/annotation.html) and RepBase for repeats. Analyses were performed with the default condition where the promoter region was set from − 1 kb to + 100 b from TSS. The positions of repeats including those CpGs on the genome were obtained from Homer_mm10_annotation_repeats files using bedtools closest utility in bedtools v2.26.0 (https://bedtools.readthedocs.io/en/latest/) and their fasta sequences were acquired from the Mus_musculus_UCSC_mm10 file (December 2011 (GRCm38/mm10) assembly of the mouse genome) using fastaFromBed in bedtools. DMRs were annotated using HOMER. Full-length L1Md sequences were obtained by L1Xplorer (http://l1base.charite.de/). Random sampling of fasta sequences were carried out using SeqKit [69]. Binding sites of transcription factors were searched for the consensus sequence of L1MdA shown in Fig. 4c using the JASPAR database (http://jaspar.genereg.net/) [70] with score threshold ≥ 90%.

### Phylogenic analysis

All L1MdA with hypo/hyperDMCs and 200 randomly selected L1MdA without any DMCs were manually annotated on Artemis genome browser (http://www.ncbi.nlm.nih.gov/pubmed/11120685) [71]. We used 33, 16, and 36 intact ORF1 sequences with hypo, hyper and no DMCs, respectively, for phylogenetic analysis. The sequences were aligned using Mafft v7.427 (http://www.ncbi.nlm.nih.gov/pubmed/18372315, November 1, 2012) [72], and manually modified on MEGA7 (https://academic.oup.com/mbe/article-lookup/doi/10.1093/molbev/msw054) [73]. Maximum-likelihood (ML) tree was inferred using IQ-TREE 1.6.10 (http://mbe.oxfordjournals.org/cgi/doi/10.1093/molbev/msu300) [74] with 100 non-parametric bootstrap replicates.

### Statistics

The differences in the methylation levels between the control and arsenic groups were tested by Student’s *t* test. Probabilities of occurrence in DMCs compared to occurrence in all CpGs were assessed by Fisher’s exact test and *p* < 0.001 was considered significant.

## Supplementary Information

**Additional file 1: Figure S1.** RRBS analysis of F1 sperm DNA that was obtained in another experiment. Number (A) and % in all CpGs (B) of hyper-/hypo-DMCs in each chromosome obtained in another independent experiment. Sperm was taken from cauda epididymis by pushed off the tissue with 26G needles in PBS, immediately transferred to a tube, and collected by centrifugation. DNA was prepared and RRBS analysis was carried out as described in the method. The sequencing was performed using an Illumina HiSeq 2500. The sequence data are publicly available at GEO with accession number GSE150500. **Figure S2.** Detailed annotation of all CpGs and hypo/hyperDMCs in all the genomic regions. **Figure S5.** Number (A) and % (B) of all CpGs classified in each chromosome using HOMER annotation. **Figure S6.** DNA methylation levels of imprinted DMRs that are methylated in the sperm. **Figure S7.** Hypo and hyperDMRs are categorized by detailed annotation as described in Method section. **Table S1.** The annotation and detailed annotation by HOMER. **Table S2**. DMRs at TSS ± 2,000 bp.

**Additional file 2: Figure S3.** Phylogenetic tree of ORF1 of L1MdA. Bootstrap support (BP) is indicated above the lines and BP < 50 are not shown. Operation taxonomy units with hypo, hyper, no DMCs are shown by blue, red, and d black colors, respectively.

**Additional file 3: Figure S4.** Phylogenetic tree of ORF2 of L1MdA. ORF2 were annotated and ML tree was inferred using the same methods of Figure S3.

## Data Availability

The datasets supporting the conclusions of this article are available in the GEO repository, accession number GSE150650 and GSE150500.
