# Supplementary material for: Gestational arsenic exposure induces site-specific DNA hypomethylation in active retrotransposon subfamilies in offspring sperm in mice
Source: Epigenetics Chromatin. 2020 Dec 2;13:53. doi: 10.1186/s13072-020-00375-3 (PMC7709384; doi:10.1186/s13072-020-00375-3)
Supplement: Supplementary file 2 — Additional file 2: Figure S3. Phylogenetic tree of ORF1 of L1MdA. Bootstrap support (BP) is indicated above the lines and BP < 50 are not shown. Operation taxonomy units with hypo, hyper, no DMCs are shown by blue, red, and d black colors, respectively. [file 13072_2020_375_MOESM2_ESM.pdf]

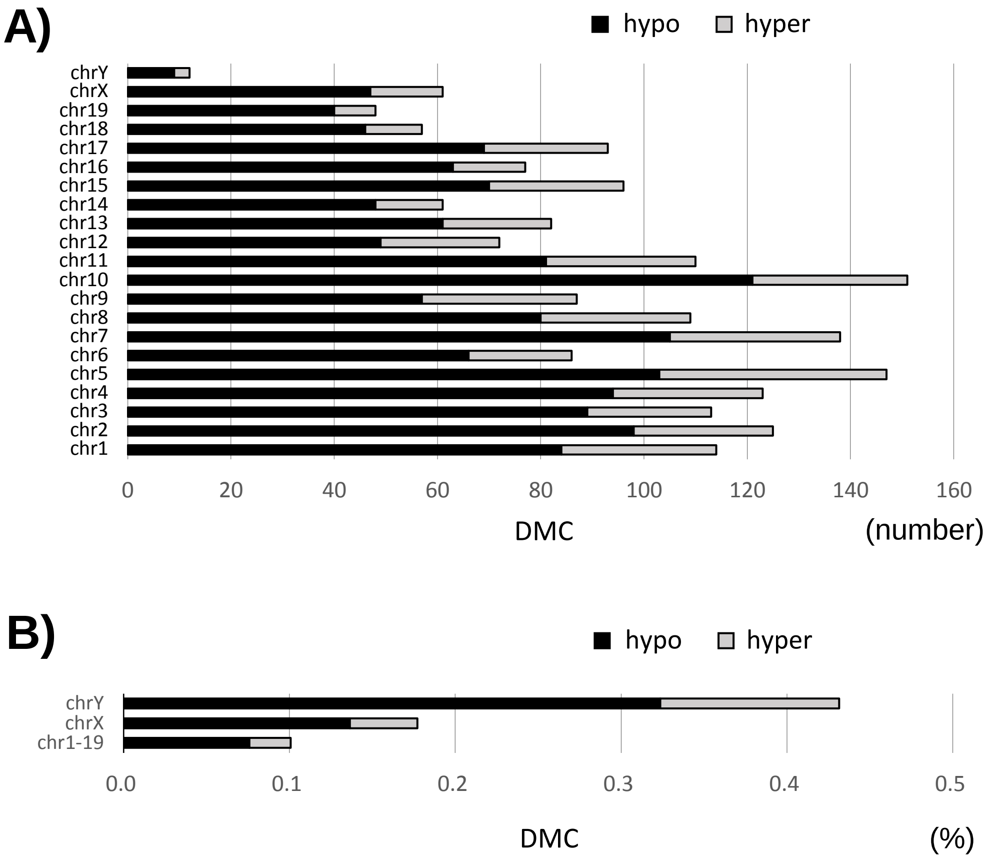

A)
hypo
hyper
chrY
chrX
chr19
chr18
chr17
chr16
chr15
chr14
chr13
chr12
chr11
chr10
chr9
chr8
chr7
chr6
chr5
chr4
chr3
chr2
chr1
0
20
40
60
80
100
120
140
160
DMC
(number)
B)
hypo
hyper
chrY
chrX
chr1-19
0.0
0.1
0.2
0.3
0.4
0.5
DMC
(%)

Figure S1.

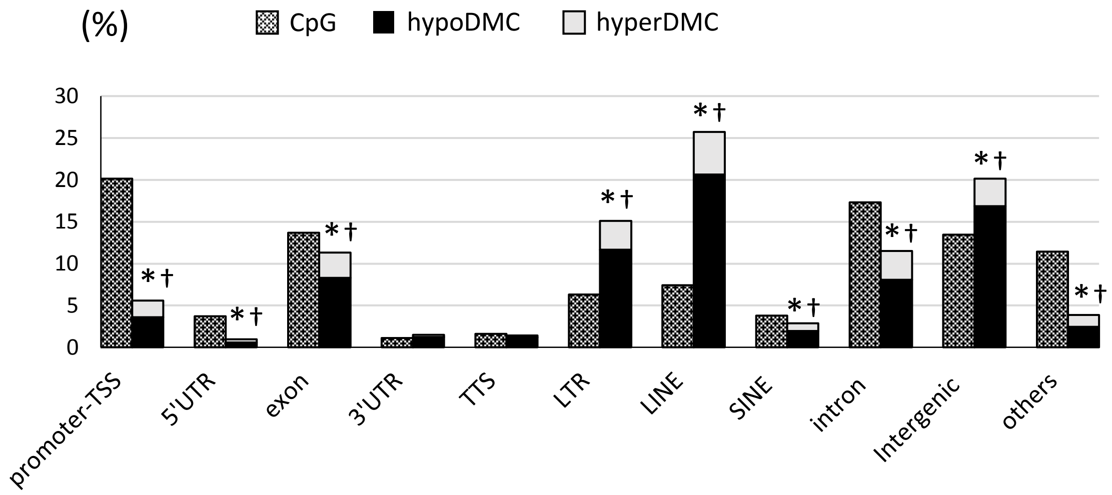

(%)
CpG
hypoDMC
hyperDMC
30
25
20
15
10
5
0
*†
*†
*†
*†
*†
*†
*†
*†
*†
promoter-TSS
5'UTR
exon
3'UTR
TTS
LTR
LINE
SINE
intron
Intergenic
others

Figure S2.

A)

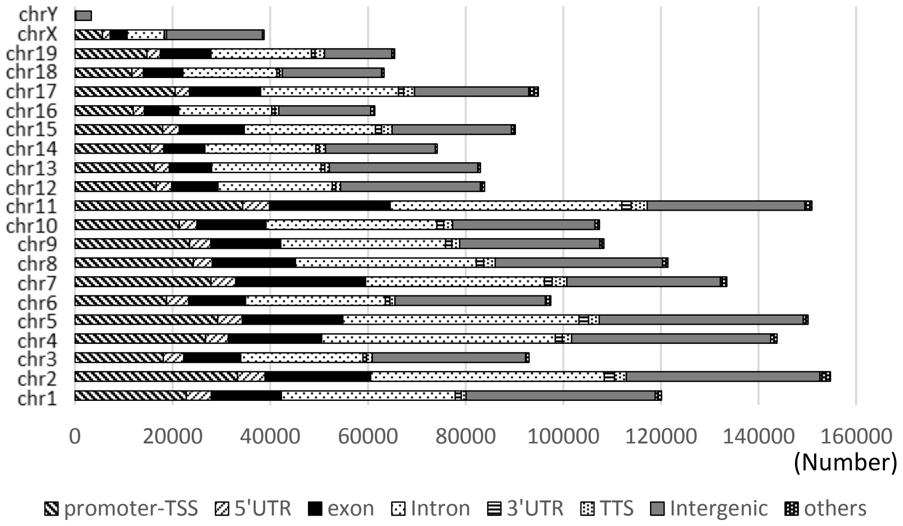
chrY
chrX
chr19
chr18
chr17
chr16
chr15
chr14
chr13
chr12
chr11
chr10
chr9
chr8
chr7
chr6
chr5
chr4
chr3
chr2
chr1
0
20000
40000
60000
80000
100000
120000
140000
160000
(Number)
promoter-TSS
5'UTR
exon
Intron
3'UTR
TTS
Intergenic
others

B)

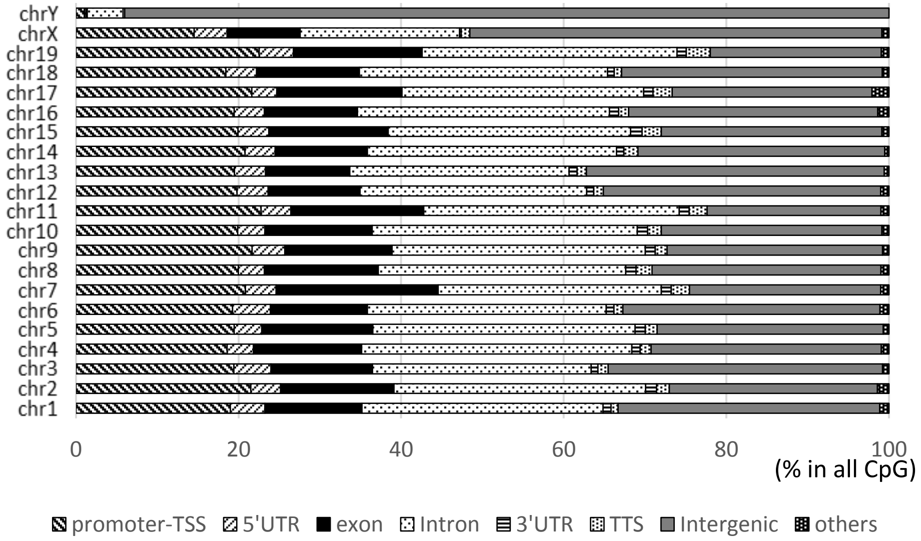
chrY
chrX
chr19
chr18
chr17
chr16
chr15
chr14
chr13
chr12
chr11
chr10
chr9
chr8
chr7
chr6
chr5
chr4
chr3
chr2
chr1
0
20
40
60
80
100
(% in all CpG)
promoter-TSS
5'UTR
exon
Intron
3'UTR
TTS
Intergenic
others

Figure S5.

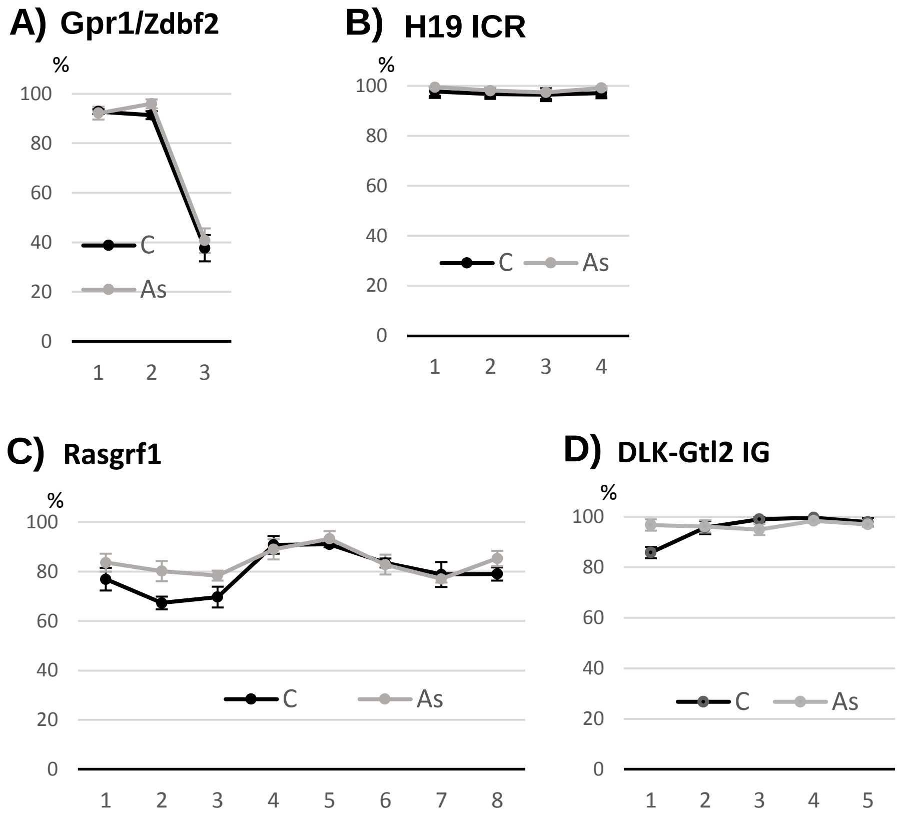
A) Gpr1/Zdbf2
%
100
80
60
40
20
0
C
As
1
2
3
B) H19 ICR
%
100
80
60
40
20
0
C
As
1
2
3
4
C) Rasgrf1
%
100
80
60
40
20
0
C
As
1
2
3
4
5
6
7
8
D) DLK-Gtl2 IG
%
100
80
60
40
20
0
C
As
1
2
3
4
5

Figure S6.

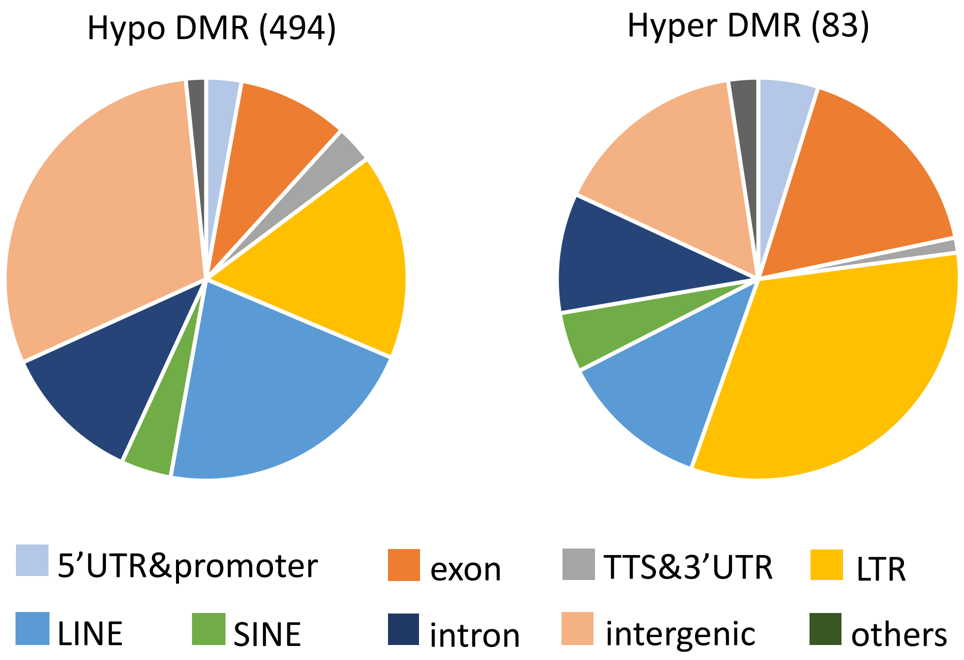
Hypo DMR (494)
Hyper DMR (83)
5’UTR&promoter
exon
TTS&3’UTR
LTR
LINE
SINE
intron
intergenic
others

Figure S7.

Table S1. The annotation and detailed annotation used by HOMER

A) The priority of annotation by HOMER[a)]

1	TSS (by default defined from -1kb to +100bp)
2	TTS (by default defined from -100 bp to +1kb)
3	CDS Exons
4	5' UTR Exons
5	3' UTR Exons
6	CpG Islands
7	Repeats
8	Introns
9	Intergenic

a) Referred from http://homer.ucsd.edu/homer/ngs/annotation.html

B) The annotation and detailed annotation used in the present study

Annotation	Detailed annotation		
	Category 1	Category 2	
Fig. 2A	Fig. 3A	Fig. 4A, 5A	Fig. 6
Promoter-TSS	Promoter-TSS	Promoter-TSS	Promoter-TSS+5'UTR
5'UTR	5'UTR	5'UTR	
Exon	Exon	Exon	Exon
Intron	LTR	IAPE	IAPE
		IAPLTR	Other LTRs
		MMERVK	
		MT	
		RLTR	
		RMER	
		LTR others	
	LINE	L1Md_A	L1Md_A
		L1Md_F	L1Md_F
		L1Md_T	L1Md_T
		L1M other	Other LINEs
		L1 other	
		LINE others	
	SINE	SINE	SINE
	Intron	Intron	Intron
	Others	Others	Others
3'UTR	3'UTR	3'UTR	TTS + 3'UTR
TTS	TTS	TTS	
Intergenic	LTR	IAPE	IAPE
		IAPLTR	Other LTRs
		MMERVK	
		MT	
		RLTR	
		RMER	
		LTR others	
	LINE	L1Md_A	L1Md_A
		L1Md_F	L1Md_F
		L1Md_T	L1Md_T
		L1M other	Other L1
		L1 other	
		LINE others	
	SINE	SINE	SINE
	Intergenic	Intergenic	Intergenic
	Others	Others	Others
Others	Others	Others	Others

Table S2. DMRs at TSS ± 2,000 bp

Chr	Start	End	Gene Name	Mean meth diff	# of CpGs	# of DMCs	Width	True distance
HypoDMC								
chr2	180227845	180227956	Lama5	-15.5	8	1	113	-1987
chr7	19406056	19406242	Klc3	-10.4	12	1	188	-1953
chr17	46784507	46784638	Rpl7l1	-15.2	9	1	133	-1852
chr17	9169474	9169673	6530411M01Rik	-15.0	4	1	201	-1453
chr16	4884877	4884922	Mgrn1	-10.7	4	1	47	-1178
chr4	150926524	150926858	Tnfrsf9	-10.6	12	1	336	-1141
chr16	30284052	30284285	Lrrc15	-10.3	10	2	235	-799
chr10	129188093	129188143	Olfr773	-14.2	3	1	52	-675
chr19	37684614	37685105	Cyp26c1	-11.7	6	1	493	-575
chr4	142017165	142017345	4930455G09Rik	-19.4	4	2	182	-553
chr3	89365583	89365751	Dcst1	-10.9	9	1	170	-331
chr5	134947017	134947141	Cldn4	-12.4	3	1	126	-84
chr11	67099211	67099742	Mir6923	-12.7	18	1	533	-62
chr10	18210942	18211067	Ect2l	-13.6	6	2	127	-53
chr13	95478680	95478750	S100z	-18.6	5	4	72	-26
chr7	126781195	126781466	Tbx6	-10.9	14	4	273	-17
chr10	25299043	25299293	Akap7	-14.8	15	9	252	0
chr11	117782283	117782448	Tmc8	-12.5	4	1	167	0
chr5	115185637	115186377	Cabp1	-12.1	12	2	742	0
chr2	38714145	38714543	Nr5a1	-11.5	9	1	400	0
chr14	54625492	54626142	Psmb11	-13.0	13	1	652	183
chr3	130617813	130617982	Etnppl	-12.7	5	2	171	239
chr5	123510995	123511206	B3gnt4	-18.3	14	8	213	536
chr17	36461236	36461329	H2-M10.4	-10.2	3	1	95	1000
chr11	102886252	102886393	Fam187a	-10.7	7	1	143	1084
chr7	126948340	126948421	Asphd1	-11.9	5	1	83	1160
chr6	116651899	116652122	8430408G22Rik	-11.6	7	2	225	1216
chr16	89959140	89959380	Tiam1	-13.1	4	1	242	1447
chr1	74793489	74793551	Wnt10a	-15.0	4	1	64	1471
chr11	70617511	70617606	Chrne	-19.8	4	2	97	1588
chr10	81498488	81498512	S1pr4	-11.5	3	1	26	1625
chr9	107677133	107677816	Gnat1	-11.9	11	4	685	1776
chr2	89787381	89787517	Olfr1254	-10.3	4	1	138	1833
HyperDMC								
chr3	95144314	95144382	Tnfaip8l2	12.9	4	1	70	-1955
chr5	142959769	142959892	Fscn1	11.1	8	2	125	-463
chr1	131743974	131744203	Slc26a9	11.8	5	1	231	0
chr4	97581962	97582054	Nfia	10.4	5	1	94	20
chr3	129754871	129755005	Egf	10.8	8	3	136	317
chr10	116986745	116986822	Best3	12.2	3	1	79	432
chr13	5862640	5862853	Klf6	10.4	3	1	215	1152
chr4	117128161	117128279	Tctex1d4	10.2	5	2	120	1349
chr9	44879591	44879909	Kmt2a	13.2	4	1	320	1365
