# Supplementary figures and images for: Gestational arsenic exposure induces site-specific DNA hypomethylation in active retrotransposon subfamilies in offspring sperm in mice
Source: Epigenetics Chromatin. 2020 Dec 2;13:53. doi: 10.1186/s13072-020-00375-3 (PMC7709384; doi:10.1186/s13072-020-00375-3)

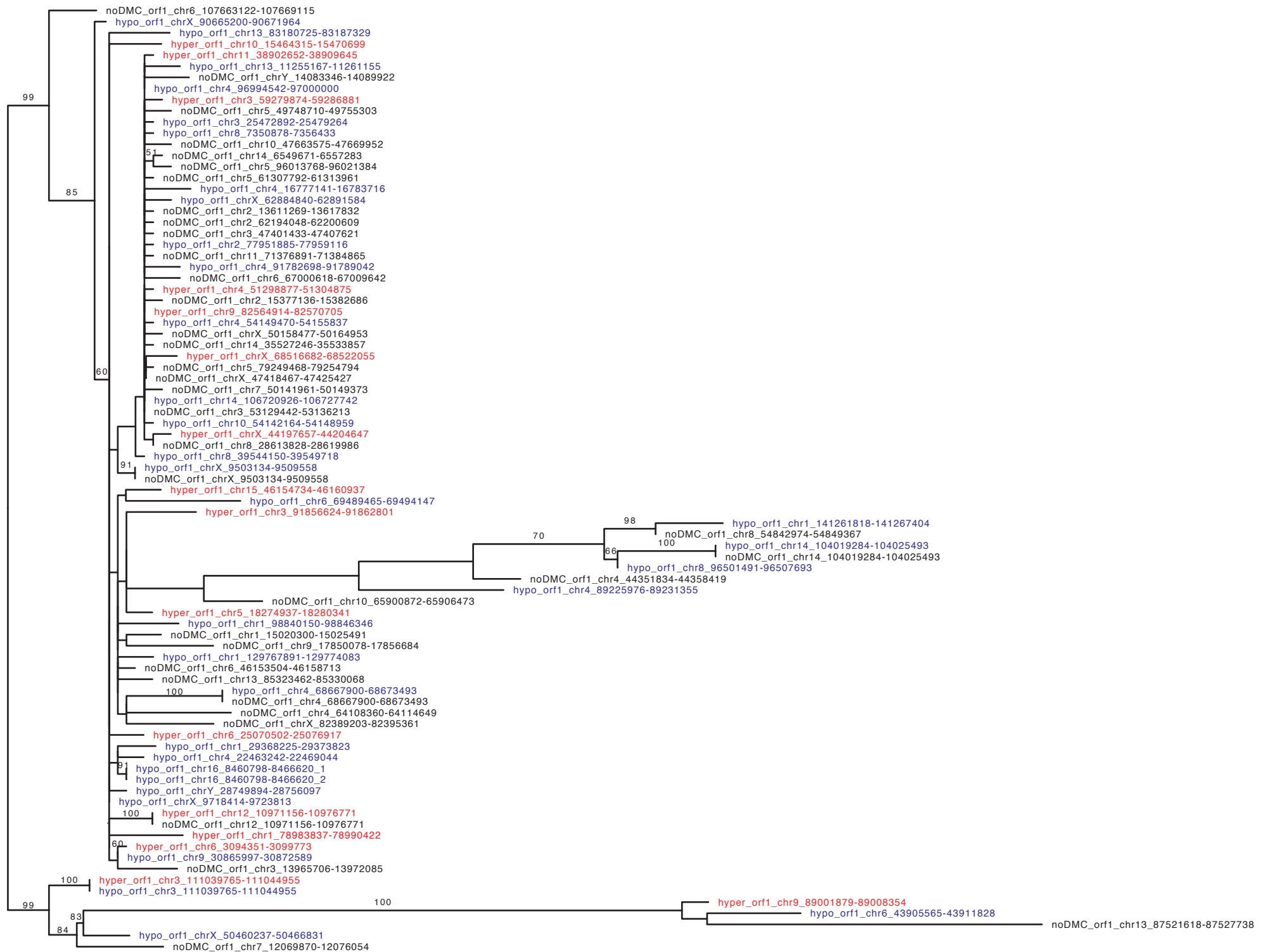

Supplement: Supplementary file 3 — Additional file 3: Figure S4. Phylogenetic tree of ORF2 of L1MdA. ORF2 were annotated and ML tree was inferred using the same methods of Figure S3. [file 13072_2020_375_MOESM3_ESM.pdf]
