# Supplementary material for: Gestational arsenic exposure induces site-specific DNA hypomethylation in active retrotransposon subfamilies in offspring sperm in mice
Source: Epigenetics Chromatin. 2020 Dec 2;13:53. doi: 10.1186/s13072-020-00375-3 (PMC7709384; doi:10.1186/s13072-020-00375-3)
Supplement: Supplementary file 1 — Additional file 1: Figure S1. RRBS analysis of F1 sperm DNA that was obtained in another experiment. Number (A) and % in all CpGs (B) of hyper-/hypo-DMCs in each chromosome obtained in another independent experiment. Sperm was taken from cauda epididymis by pushed off the tissue with 26G needles in PBS, immediately transferred to a tube, and collected by centrifugation. DNA was prepared and RRBS analysis was carried out as described in the method. The sequencing was performed using an Illumina HiSeq 2500. The sequence data are publicly available at GEO with accession number GSE150500. Figure S2. Detailed annotation of all CpGs and hypo/hyperDMCs in all the genomic regions. Figure S5. Number (A) and % (B) of all CpGs classified in each chromosome using HOMER annotation. Figure S6. DNA methylation levels of imprinted DMRs that are methylated in the sperm. Figure S7. Hypo and hyperDMRs are categorized by detailed annotation as described in Method section. Table S1. The annotation and detailed annotation by HOMER. Table S2. DMRs at TSS ± 2,000 bp. [file 13072_2020_375_MOESM1_ESM.pdf]

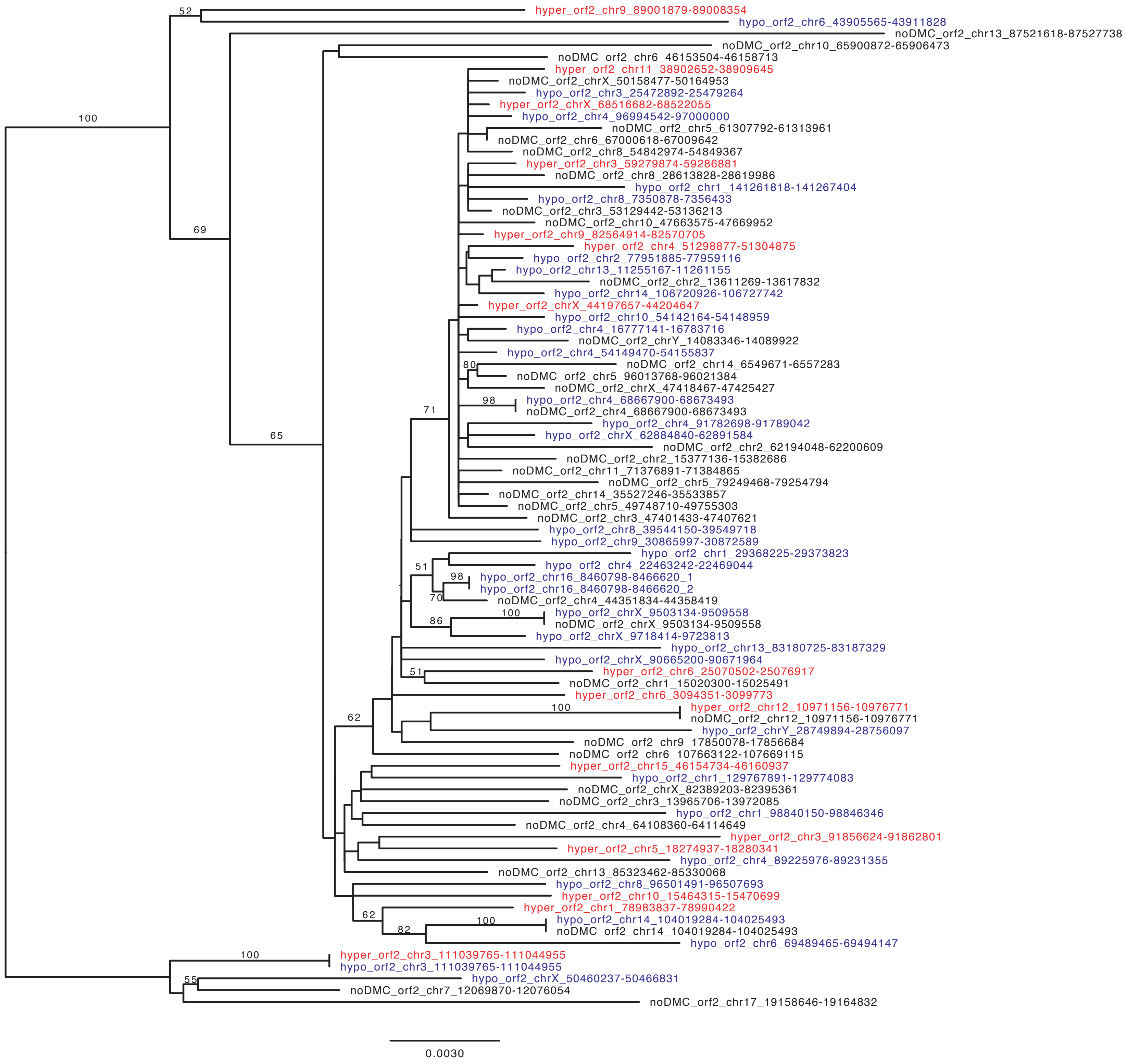
hyper_orf2_chr9_89001879-89008354
hypo_orf2_chr6_43905565-43911828
noDMC_orf2_chr13_87521618-87527738
noDMC_orf2_chr10_65900872-65906473
noDMC_orf2_chr6_46153504-46158713
hyper_orf2_chr11_38902652-38909645
noDMC_orf2_chrX_50158477-50164953
hypo_orf2_chr3_25472892-25479264
hyper_orf2_chrX_68516682-68522055
hypo_orf2_chr4_96994542-97000000
noDMC_orf2_chr5_61307792-61313961
noDMC_orf2_chr6_67000618-67009642
noDMC_orf2_chr8_54842974-54849367
hyper_orf2_chr3_59279874-59286881
noDMC_orf2_chr8_28613828-28619986
hypo_orf2_chr1_141261818-141267404
hypo_orf2_chr8_7350878-7356433
noDMC_orf2_chr3_53129442-53136213
noDMC_orf2_chr10_47663575-47669952
hyper_orf2_chr9_82564914-82570705
hyper_orf2_chr4_51298877-51304875
hypo_orf2_chr2_77951885-77959116
hypo_orf2_chr13_11255167-11261155
noDMC_orf2_chr2_13611269-13617832
hypo_orf2_chr14_106720926-106727742
hyper_orf2_chrX_44197657-44204647
hypo_orf2_chr10_54142164-54148959
hypo_orf2_chr4_16777141-16783716
noDMC_orf2_chrY_14083346-14089922
hypo_orf2_chr4_54149470-54155837
noDMC_orf2_chr14_6549671-6557283
noDMC_orf2_chr5_96013768-96021384
noDMC_orf2_chrX_47418467-47425427
hypo_orf2_chr4_68667900-68673493
noDMC_orf2_chr4_68667900-68673493
hypo_orf2_chr4_91782698-91789042
hypo_orf2_chrX_62884840-62891584
noDMC_orf2_chr2_62194048-62200609
noDMC_orf2_chr2_15377136-15382686
noDMC_orf2_chr11_71376891-71384865
noDMC_orf2_chr5_79249468-79254794
noDMC_orf2_chr14_35527246-35533857
noDMC_orf2_chr5_49748710-49755303
noDMC_orf2_chr3_47401433-47407621
hypo_orf2_chr8_39544150-39549718
hypo_orf2_chr9_30865997-30872589
hypo_orf2_chr1_29368225-29373823
hypo_orf2_chr4_22463242-22469044
hypo_orf2_chr16_8460798-8466620_1
hypo_orf2_chr16_8460798-8466620_2
noDMC_orf2_chr4_44351834-44358419
hypo_orf2_chrX_9503134-9509558
noDMC_orf2_chrX_9503134-9509558
hypo_orf2_chrX_9718414-9723813
hypo_orf2_chr13_83180725-83187329
hypo_orf2_chrX_90665200-90671964
hyper_orf2_chr6_25070502-25076917
noDMC_orf2_chr1_15020300-15025491
hyper_orf2_chr6_3094351-3099773
hyper_orf2_chr12_10971156-10976771
noDMC_orf2_chr12_10971156-10976771
hypo_orf2_chrY_28749894-28756097
noDMC_orf2_chr9_17850078-17856684
noDMC_orf2_chr6_107663122-107669115
hyper_orf2_chr15_46154734-46160937
hypo_orf2_chr1_129767891-129774083
noDMC_orf2_chrX_82389203-82395361
noDMC_orf2_chr3_13965706-13972085
hypo_orf2_chr1_98840150-98846346
noDMC_orf2_chr4_64108360-64114649
hyper_orf2_chr3_91856624-91862801
hyper_orf2_chr5_18274937-18280341
hypo_orf2_chr4_89225976-89231355
noDMC_orf2_chr13_85323462-85330068
hypo_orf2_chr8_96501491-96507693
hyper_orf2_chr10_15464315-15470699
hyper_orf2_chr1_78983837-78990422
hypo_orf2_chr14_104019284-104025493
noDMC_orf2_chr14_104019284-104025493
hypo_orf2_chr6_69489465-69494147
hyper_orf2_chr3_111039765-111044955
hypo_orf2_chr3_111039765-111044955
hypo_orf2_chrX_50460237-50466831
noDMC_orf2_chr7_12069870-12076054
noDMC_orf2_chr17_19158646-19164832
52
100
69
65
71
80
98
51
98
70
86
100
51
100
62
62
82
100
100
55
0.0030
